# Exploring Factors That Affected Student Well-Being during the COVID-19 Pandemic: A Comparison of Data-Mining Approaches

**DOI:** 10.3390/ijerph191811267

**Published:** 2022-09-07

**Authors:** Hülya Yürekli, Öyküm Esra Yiğit, Okan Bulut, Min Lu, Ersoy Öz

**Affiliations:** 1 Department of Statistics, Yıldız Technical University, Istanbul 34220, Türkiye; 2 Centre for Research in Applied Measurement and Evaluation, University of Alberta, Edmonton, AB T6G 2G5, Canada; 3 Department of Public Health Sciences, Miler School of Medicine, University of Miami, Miami, FL 33136, USA

**Keywords:** student well-being, data mining, educational disruption, COVID-19, school closures

## Abstract

COVID-19-related school closures caused unprecedented and prolonged disruption to daily life, education, and social and physical activities. This disruption in the life course affected the well-being of students from different age groups. This study proposed analyzing student well-being and determining the most influential factors that affected student well-being during the COVID-19 pandemic. With this aim, we adopted a cross-sectional study designed to analyze the student data from the Responses to Educational Disruption Survey (REDS) collected between December 2020 and July 2021 from a large sample of grade 8 or equivalent students from eight countries (*n* = 20,720), including Burkina Faso, Denmark, Ethiopia, Kenya, the Russian Federation, Slovenia, the United Arab Emirates, and Uzbekistan. We first estimated a well-being IRT score for each student in the REDS student database. Then, we used 10 data-mining approaches to determine the most influential factors that affected the well-being of students during the COVID-19 outbreak. Overall, 178 factors were analyzed. The results indicated that the most influential factors on student well-being were multifarious. The most influential variables on student well-being were students’ worries about contracting COVID-19 at school, their learning progress during the COVID-19 disruption, their motivation to learn when school reopened, and their excitement to reunite with friends after the COVID-19 disruption.

## 1. Introduction

The global pandemic caused by COVID-19 has disrupted education across the globe. During the pandemic, many local governments around the world ordered school closures to help mitigate the transmission and spread of the virus. Although closing schools resulted in more time to spend with family members [1,2,3], it also led to significant disruptions of daily and school routines, lack of access to school-based resources, prolonged periods of social isolation [4], less time spent outdoors [5], and other COVID-19-related stressors, such as COVID-19 infection risk and family economic adversity [6,7,8]. Therefore, significant concerns were raised over pandemic-related school closures’ effects on students’ well-being.

The COVID-19 pandemic led to an extremely extensive educational disruption that has affected 94% of the students in the world not only in terms of education, but also economically, socially, and psychologically [9]. This disruption in education caused both mental and physical health issues for youth and caused a decrease in learning gains, especially for those students of color, students with disabilities, English language learners, and low-income students [10]. Teachers also experienced a medium-to-high amount of stress and encountered obstacles such as excessive workloads, lack of access to computer hardware, and low student motivation during the COVID-19 lockdowns [11], which caused a significant decline in people’s mental health [12].

Well-being is “a dynamic state characterized by students experiencing the ability and opportunity to fulfill their personal and social goals” and it consists of five domains: cognitive, psychological, physical, social, and material [13] (p. 8). Positive well-being in adolescents is associated with better-perceived general health and fewer risky health behaviors during young adulthood [14]. Since students’ well-being is essential to their development, it is important to reveal the factors that affected student well-being during the COVID-19 outbreak.

A student’s well-being can be assessed through several methods such as interviews with the student, standardized tests, and single-item questions from nationwide data [15]. Several studies examined the well-being of higher-education students globally [16,17] and within countries [18,19,20,21,22] and investigated adolescents’ [23] and graduate students’ [24] well-being in different countries during the COVID-19 pandemic via online surveys. However, less is known about the impact of the COVID-19 pandemic on the well-being of secondary-education-level students living around the world. Thus, the present work used a broad database consisting of grade 8 or equivalent students to reveal the factors that potentially impacted students’ well-being throughout the world.

Data-mining techniques have been widely used in many scientific disciplines to put forth the existing relations and explore the hidden patterns in large-scale data [25] that contain extensive information. The popularity of data-mining approaches stems from their ability to deal with data that is non-normal, heterogeneous, and nonlinear, which are violations of some assumptions in traditional statistical methods. In the educational environment, the use of the data-mining methods has a special name educational data mining (EDM), which aims to develop methods for educational data to obtain a better understanding of students and their learning environments [26]. The term EDM was firstly studied in detail in a review study by Romero and Ventura [27] and then extended by the same authors in 2010 and 2013 [26,28]. In addition, review studies by Baker and Yacef [29], Peña-Ayala [30], Dutt et al. [31], Bakhshinategh et al. [32], Aldowah et al. [33], and Namoun and Alshanqiti [34] presented different applications in the EDM literature.

The outputs obtained from EDM provide valuable information to policymakers, especially in efforts to improve teaching and learning quality. The rapidly growing trend in the EDM field is highly associated with the availability and accessibility of large data sources, especially assessment studies conducted at international levels such as Trends in International Mathematics and Science Study (TIMSS). As stated by both Bakhshinategh et al. [32] and Hernandez-Blanco et al. [35], EDM methods are applied for different tasks such as student modeling (e.g., predicting students’ performance, detecting undesirable behaviors, profiling/grouping students, and social network analysis), decision support systems (e.g., providing feedback, creating alerts and helping in planning/scheduling for stakeholders, creating courseware, developing concept maps, and generating recommendation) and other applications (e.g., adaptive systems, evaluation, and scientific inquiry). This paper aimed to use various data-mining approaches to explore the variables that influenced student well-being during the COVID-19 pandemic. So far, no study has combined EDM techniques with student well-being during COVID-19.

## 2. Materials and Methods

### 2.1. Data

We used the Responses to Educational Disruption Survey (REDS) to reveal the factors that potentially affected students’ well-being. The United Nations Educational, Scientific and Cultural Organization (UNESCO), in collaboration with the International Association for Evaluation of Educational Achievement (IEA) and the European Commission, initiated the REDS study to identify the effects of the pandemic on teaching and learning, and to investigate the responses of education stakeholders to the educational disruption [36]. The REDS database provides information on several themes, including the well-being of students and teachers, students’ academic progress, and the government’s responses to the disruption in education during the COVID-19 outbreak in different countries [36]. REDS student data were collected via a student questionnaire that included 40 questions covering how teaching and learning changed due to COVID-19; student well-being; students with special needs; schooling after the COVID-19 disruption; persisting influences of COVID-19; and demographic information on the students, their parents, and their homes [37]. All questions in the REDS student questionnaire are listed in the Supplementary Materials (see Table S1) and can also be found in the REDS user guide [37]. REDS questionnaire development was organized by the IEA and led by the Australian Council for Educational Research (ACER) along with experts from UNESCO, the European Commission Joint Research Centre (JRC), the IEA, and the participating countries [36].

REDS investigated the effects of the COVID-19 pandemic on teaching and learning and the education stakeholders’ responses to the disruption in education within and across 11 countries [36]. REDS collected data from schools, teachers, and grade 8 or equivalent students. Student-level data were collected from eight countries, including Burkina Faso, Denmark, Ethiopia, Kenya, the Russian Federation, Slovenia, the United Arab Emirates, and Uzbekistan. The data were collected between December 2020 and July 2021. During this period, at least one physical school closure occurred due to COVID-19 in all participating countries [36]. The REDS student questionnaire included 40 questions consisting of several topics, statements, and situations that are referred to as “items” in general from here on. The student questionnaire was administered in both online and paper–pencil formats.

#### Measures for Student Well-Being

The REDS student questionnaire included a separate well-being section with four questions (Questions 23, 24, 25, and 26) that measured students’ well-being [37]. Question 23 asked whether the school or teachers gave the students information to support student well-being during the COVID-19 disruption. Several topics such as “looking after my emotional well-being”, “looking after my personal safety”, and “healthy eating” were listed, and students were asked to mark one of the three choices: yes and it was helpful, yes but it was not helpful, or no. Question 24 regarded the extent of agreeing or disagreeing with several statements about how the students felt during the COVID-19 disruption. Some of these statements were “I felt anxious about the changes in my schooling”, “I felt overwhelmed by what was happening in the world due to the COVID-19 pandemic”, and “I felt overwhelmed by what was happening in my local area due to the COVID-19 pandemic”. The responses used a four-point Likert-type scale (i.e., strongly disagree, disagree, agree, and strongly agree). Question 25 regarded the extent of agreeing or disagreeing with some statements about student well-being during the COVID-19 disruption. The statements included “I exercised (including walking) more than usual”, “I was able to do more of my usual outside of school activities (scouts, guides, training for sports)”, and “I felt fit and healthy” among others. This question was answered using the same four-point Likert-type scale: strongly disagree, disagree, agree, and strongly agree. Finally, Question 26 asked whether the student was affected by any listed situation during the COVID-19 pandemic. A few of these situations were “one or both of my (parents/guardians) lost their job”, “our family had to be more careful with money than usual”, and “one or both of my (parents/guardians) were stressed about their job”. Question 26 was a yes-or-no question. Since Question 24 asked how the students felt during the COVID-19 disruption and Question 25 asked about the students’ well-being, and since both questions 24 and 25 used a four-point Likert scale, only these two questions were used to assess student well-being in this study. The questions are listed in the Supplementary Materials (Table S1). 

First, REDS student data files for all countries were merged and country-specific items were removed. Questions 24 and 25 in the REDS student questionnaire consisted of 23 items that measured student well-being during the COVID-19 outbreak. Among these items, responses to the negatively worded items were reverse coded to align all items in the same direction (i.e., positive wording). Items with more than 30% missing values were removed, and all the remaining items were used in the analysis. In addition, students who did not respond to all items of Questions 24 and 25 in the student questionnaire well-being section were removed from the study.

To deal with the missingness in the REDS student dataset, two different data files were used: the first dataset was the original data with the missing cases (DM), and a second dataset was created using multiple imputation (DMI). A multiple imputation procedure was used to consider the missing values. The DMI dataset was created using multiple imputation with chain equations utilizing classification and regression trees (MICE-CART) due to its high performance and low computational cost [38].

Next, using the 23 items in both datasets, an exploratory factor analysis (EFA) was conducted using Mplus version 7 [39]. Since we were handling categorical data, the weighted least squares mean and variance adjusted estimator was used with CF-quartimax (oblique) rotation. Through factor analysis, four factors were extracted. Then, in order to obtain the student well-being scores, a bifactor graded response model was estimated using the DM and DMI datasets. Finally, based on their well-being item response theory (IRT) scores, the students were classified into two categories: students whose well-being score was below the average well-being score of all students in the database and students whose well-being score was above the average well-being score of all students in the database.

### 2.2. Predictive Data-Mining Techniques

Among different supervised classification techniques, some classifiers such as *k*-nearest neighbor (k-NN), decision tree (DT), support vector machines (SVMs), and logistic regression (LR) are widely used in the EDM literature. Except for random forest (RF), ensemble-learning-based methods such as adaptive boosting (AdaBoost), the gradient boosting machine (GBM), eXtreme gradient boosting (XGBoost), the light gradient boosting machine (LightGBM), and categorical boosting (CatBoost) have been more rarely used in the literature. Regardless of the frequency of using the mentioned learning methods, it should be noted that according to the “No-Free-Lunch” theorem, there is no single superior classifier that perfectly fits every dataset. Therefore, a comprehensive pipeline development such as parameter optimization and variable selection is required for such data-specific techniques [40]. Once the models are built, some fundamental properties (i.e., predictive accuracy, speed, robustness, scalability, interpretability, and simplicity [41]) assist in the selection of the final model that will be used for different tasks, as stated in Bakhshinategh et al. [32] and Hernandez-Blanco et al. [35]. The following subsections introduce the EDM application strategies utilized in this study. 

#### 2.2.1. Methods Used for Classification

The current study aimed to explore factors that affected students’ well-being during the COVID-19 pandemic using a comprehensive comparative evaluation of four single and six ensemble learners (ELs) simultaneously.

##### *k*-NN

The *k*-NN is one of the instance-based classifiers, meaning it is based on the distance measure of instances in the training data for which class labels are already known [42]. An unlabeled instance (observation) searches for the *k*-nearest neighbors; i.e., the most similar instances, based on predetermined distance measures such as Euclidean. Since there is no training phase (calculations are performed during inference), *k*-NN is considered purely lazy. The classification performance depends on the choice of the single parameter of the model, *k*.

##### Decision Tree

The primary purpose of a DT is to divide a dataset containing a large number of records into smaller, more understandable subgroups by applying specific rules with a divide-and-conquer strategy and create a prediction by learning the simple decision rules derived from the given information [43].

##### Random Forest

RF [44] is one of the most popular ensemble learners for classification and regression purposes. The primary goal is to create a forest consisting of several DTs. Each tree in the forest is built with a different subset of the training instances with random selection to reduce the variance in results and increase the accuracy in classification.

##### Logistic Regression

LR [45] is used to model the dependent variable with a binary measurement level. In LR, a classification model is constructed with the instances in the training data and unlabeled instances are assigned to a class with the highest probability. Another purpose of the LR method is to search for the variables that significantly affect the dependent variable.

##### Support Vector Machines

SVMs are supervised machine learners that can classify linear and nonlinear data [46]. The nonlinear SVM is used to make a classification when the data cannot be linearly separated. The transformation of nonlinear space to linear is utilized with a specific kernel function such as linear, polynomial, or radial basis (RBF). It is intended to find the hyperplane where labels are separated with the maximum margin. The classification performance is associated with the choice of kernel function and with the model’s parameter settings. RBF, the most efficient kernel function in SVM, was used in this study.

##### Boosting Algorithms

This study utilized RF, AdaBoost, GBM, XGBoost, LightGBM, and CatBoost as ELs in which a set of classifiers were trained and combined to obtain a better performance and possibly overcome some limitations of a single learner, such as the existence of complex, noisy, and high-dimensional structured independent variables and an imbalanced dependent variable [47,48]. The basic idea of ELs is to combine multiple weak learners to obtain a much more powerful learner with maximum performance. Based on the hypothesis that the performance of combined weak learners produces better accuracy than a single learner with the same training size, ELs have recently become a frequently used machine learning (ML) application. In addition, most ML models require a careful preprocessing procedure for the dataset with a high proportion of missing values before the training phase. On the other hand, some boosting algorithms (e.g., XGBoost, LightGBM, and CatBoost) can handle missing values internally without observation deletion or value imputation. Despite their promising prediction performance, high generalization accuracy, and high computational capability due to the parallel processing, only a few studies have investigated the ELs in EDM literature. Further, none of the previous studies on international large-scale assessment surveys such as TIMSS provided a comprehensive evaluation for boosting algorithms.

Adaptive boosting (Adaboost) was the first efficient boosting algorithm [49] for binary classification. A single-layer DT is used as a weak classifier (e.g., decision stump). In the first step, the weight of each sample is initialized. The second step involves updating the weights according to the classification performance of the previous iteration. The weights of misclassified samples are increased to make them more important in the next iteration. In other words, more weight is given to the misclassified samples to obtain the correct classification in the subsequent decision stump. In the third step, the ensemble weights of weak classifiers are computed according to their performances. The second step is reiterated until the maximum iteration level is reached or all the samples are correctly classified.

While the weights of wrongly classified samples are under consideration in Adaboost, gradient boosting (GB)-type algorithms attempt to optimize the loss function. Here, the learning process is based on residual errors of the previous classifier. The training phase in GBM is utilized in a stagewise process. The training of several models is gradual, additive, and sequential [50]. At each step, the GBM iteratively adds a new DT to reduce the loss function while the trees added to the model in the previous steps remain unmodified.

XGBoost is a type of GB designed to be highly scalable [50,51]. XGBoost supports parallel processing in tree construction to speed up the training. In XGBoost, a gradient learning strategy is employed by weak learners. When including an additional regularization term for the loss function (shrinkage), a better classification performance can be achieved with a higher speed with XGBoost compared to GB. In addition, the advanced regularization helps to deal with overfitting problems and controls the complexity.

As in XGBoost, LightGBM [52] also approximates the loss function by utilizing second-order Taylor expansion; the complexity is controlled by the regularization term. However, the essential difference between the two boosting methods is the procedure used in tree growth. LightGBM uses a leafwise (vertical) growth instead of the levelwise (horizontal) growth used by XGBoost. Therefore, the increase in speed and decrease in memory consumption without sacrificing performance have recently made LightGBM more preferable for EL applications [53]. However, LightGBM requires careful hyperparameter tuning and is prone to overfitting for training samples with a small size.

CatBoost is an open-source ML library that can internally distinguish the different levels of categorical independent variables without preprocessing such as one-hot encoding. When the dataset has many categorical variables that possess a high cardinality (too many unique levels), one-hot encoding leads to memory consumption and a curse of dimensionality problems. Therefore, CatBoost was proposed to tackle these problems. It uses a random permutation mechanism for calculating leaf values [54].

#### 2.2.2. Variable Selection and Importance

The performance of most ML models largely depends on the variables selected as inputs. Variables selected as inputs and then fed to the learners are categorized into two types: (1) relevant variables that increase the model’s performance when included and cause information loss when excluded; and (2) irrelevant variables that do not contribute to the model’s performance when included and do not cause information loss when excluded.

It is very important to strike a balance between using all of the relevant variables and, at the same time, constructing the simplest model. Variable selection techniques are used to form a subset of variables to avoid the “curse of dimensionality”, which often leads to overfitting and decreasing the computational cost [55,56]. Additionally, the physical meaning of the variables in the original data is preserved and better interpretability is provided [57,58,59]. In this study, the initial variable space had hundreds of items. Some were not informative due to their irrelevant or redundant structure [60] on the dependent variable; in our case, a well-being score below and above the average. Therefore, reducing the initial variable space and building models with a subset of items, including the most important ones, had to be handled. For this purpose, a two-step procedure in which multiple variable selection methods were initially considered to capture both the linear and nonlinear associations was utilized. Then, their results with a simple ensemble-based fusion strategy were combined. In the first step, ranked variables according to their importance scores were obtained using three variable selection methods: the feature importance function, the SHapley Additive exPlanations (SHAP) function, and the mutual information ranker.

An importance score of a variable refers to how strongly the relevant variable can discriminate between two classes of the dependent variable. It is worth mentioning that the influential variables on well-being suggested by each variable selection method and their effect sizes were different. The Borda method was used because it provided the opportunity to make a fair selection by considering the output of each variable selection method simultaneously to obtain a general ranking. Therefore, the second step was carried out to combine variable selection methods using the Borda count procedure [61]. This procedure is based on variables’ relative ranking; the scores produced by different rankers do not require normalization within the same range [62]. The following equation was used to calculate the Borda scores:

(1) $$B\left(c\right)=\underset{j}{\sum }B_{j}\left(c\right),$$

where *j* is the ranker and *B_j_*(*c*) shows the rank of the variable obtained by *j*th ranker. When Borda scores are sorted in descending order, the final ranking of variables from best to worst is obtained.

#### 2.2.3. Performance Evaluation and Data-Mining Procedure

For each learner, a composite procedure [63] based on *k*-fold cross-validation (CV) and grid search (GS) was applied to identify the optimal hyperparameters that provided the maximum accuracy score in the estimation. In each learner, the hyperparameter configuration search space was defined and the composite procedure was applied for the training subset constructed using random sampling from the data. With the optimal hyperparameters determined by working on the training, the learning models were rerun on the test set and the classification results were obtained.

The performance benchmarking of the selected classifiers was carried out with an accuracy (ACC) metric used in almost all classification problems. Based on the confusion matrix that gives the summative information about how well any learner classifies the tuples of classes, the ACC metric (the percentage of correctly classified samples) was calculated using the following equation:

(2) $$\mathrm{ACC}=\frac{\mathrm{Number}\;\mathrm{of}\;\mathrm{correct}\;\mathrm{predictions}}{\mathrm{Total}\;\mathrm{number}\;\mathrm{of}\;\mathrm{predictions}}=\frac{\mathrm{TP}+\mathrm{TN}}{\mathrm{TP}+\mathrm{FN}+\mathrm{FP}+\mathrm{TN}},$$

where TP (true positive) is the correct positive prediction, FP (false positive) is the incorrect positive prediction, TN (true negative) is the correct negative prediction, and FN (false negative) is the incorrect negative prediction.

In summary, since the primary purpose of this study was to explore the most influential variables in classifying students (i.e., whether their well-being scores were above or below the average), the steps of the statistical analysis for achieving the mentioned purpose using single and ensemble learners are given below:

- Step 1. Data split. Both datasets (DM and DMI) were divided into training and testing at two different proportions (20% and 30%). First, the learners were trained with the training set, and then the classification performance of each learner was tested with the test set.
- Step 2. Variable selection. Firstly, a set was created with all of the variables handled in this study. Then, a two-step procedure was utilized. Variable importance scores and ranks were obtained using three different variable selection methods (the mutual information, the feature importance function, and SHAP). Note that each selection method has its own application ability. For example, the mutual information function only works with the full information; in other words, it cannot handle data with missing values. Therefore, the mutual information method was the only method used for the DM dataset. On the other hand, the feature importance function is performed with a prespecified learner. XGBoost, LightGBM, and CatBoost learners were used for the DM dataset. For the DMI dataset, XGBoost, LightGBM, CatBoost, AdaBoost, GBM, LR, and RF were used. Finally, as the last variable selection method, variable scores were obtained according to the different learners of the SHAP function. XGBoost, LightGBM, and CatBoost algorithms were used for the DM dataset and XGBoost, LightGBM, CatBoost, and GBM algorithms were used for the DMI dataset. The Borda count procedure was then applied in the rank aggregation. Thus, a list of variables from the most influential to the least influential was created. Then, the top 10%, 20%, 30%, 40%, and 100% (all) of the variables were selected as the inputs for the learners.
- Step 3. Classification. After variable selection, the classification step was achieved. Each learner had specific and different numbers of hyperparameters that had to be tuned. According to the predefined hyperparameter configuration search space, the composite procedure based on *k*-fold CV and GS was applied for each learner. Since the choice of the number of folds depended on different factors such as the training sample size and the number of tuning parameters, there was no strictly defined rule. However, as Jung [64] suggested, we set the fold number *k* at 5. The learning models were rerun on the testing data using the optimal hyperparameters and the results for their corresponding performance metrics were compared.

## 3. Results

The REDS student questionnaire consisted of 40 questions related to demographic characteristics, students with special needs, teaching and learning changes, students’ well-being, returning to school after the COVID-19 pandemic’s disruption, and influences that persisted during the COVID-19 outbreak [37]. The participants of this study were eighth-grade or equivalent students from all eight countries (Burkina Faso, Denmark, Ethiopia, Kenya, the Russian Federation, Slovenia, the United Arab Emirates, and Uzbekistan) that participated in the REDS student questionnaire. The target student population for the REDS was all the students in their eighth year of schooling [37]. The mean age of the students was 14.2 (*SD* = 1.2). The final sample included 20,720 students.

### 3.1. The Student Well-Being Score

**Figure 1:**
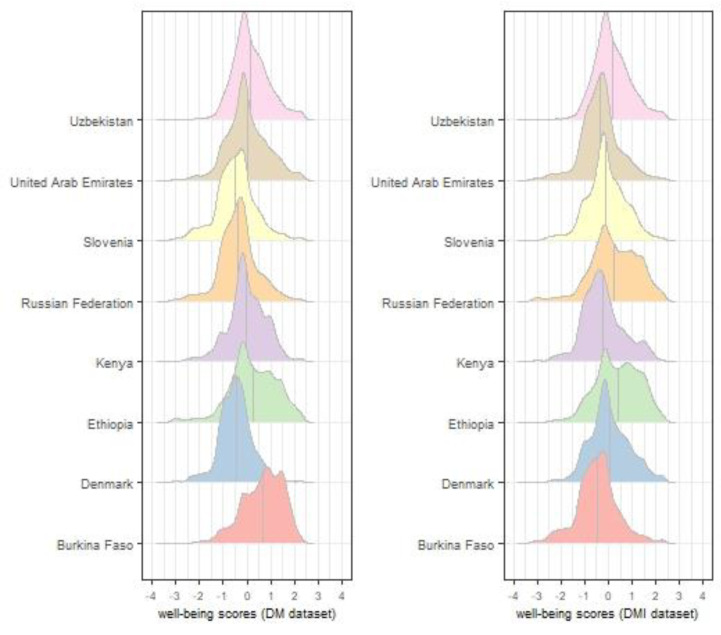
Distribution of well-being scores using DM and DMI datasets.

EFA was conducted and the results were similar for the DM and DMI datasets. Four factors were extracted by the EFA using both datasets. Two items (“I could not get my usual level of support from non-teaching support staff” and “I used social media a lot more than before the COVID-19 disruption”) were removed due to low (lower than 0.3) and negative factor loadings. Then, a second EFA was conducted using the remaining 21 items. The root mean square error of approximation (RMSEA), comparative fit index (CFI), and Tucker–Lewis index (TLI) values for the DM dataset were 0.06, 0.955, and 0.928, respectively. For the DMI dataset, the RMSEA was 0.06, the CFI was 0.954, and the TLI was 0.926. Through factor analyses, four factors were extracted. Finally, a bifactor graded response model was estimated using both datasets and student well-being IRT scores were obtained. The mean and standard deviation (*SD*) of the student well-being score for each country is listed in Table 1. In addition, the well-being score distributions for each country for the DM and DMI datasets are shown in Figure 1. Once the dependent variable of well-being was created, it was classified into two categories: well-being above the mean well-being score of all students in the database and well-being below the mean well-being score of all students in the database.

### 3.2. Classification

#### 3.2.1. Classification Performed Using the DM Dataset

Since the CatBoost, LightGBM, and XGBoost algorithms can work with missing values, they were used to classify students’ well-being in the DM dataset. First, classifications were performed on the 20% and 30% testing sets using all the variables. Next, six different scores were obtained for the variables using the XGBoost, LightGBM, and CatBoost algorithms within the feature importance and SHAP functions. The variables were ordered from the highest to the lowest explanatory value by evaluating these scores using the Borda method. By using the top 10% (top 18 variables), 20% (top 36 variables), 30% (top 54 variables), and 40% (top 72 variables) of the ranked variables, classifications were performed on the 20% and 30% testing sets. The accuracy values for these classifications are given in Table 2.

According to the data in Table 2, the CatBoost algorithm was the most successful classifier, with an accuracy of 77.059% in the 30% test set. In comparison, the LightGBM algorithm reached an accuracy of 77.051% in the 20% test set in the classification using all the variables. When the top 18 variables with the highest explanatory power were used in the classification, the CatBoost and LightGBM algorithms performed similarly, with an accuracy of almost 76% in the 20% test set. All classification algorithms used in this research returned similar results for the 30% and 20% test sets considering all variable selection algorithms. In particular, compared with using all the variables in the classification process (classification without variable selection), the classification achieved with reduced variable space (classification with variable selection) produced similar and sometimes better accuracy values. Therefore, it was shown that the variable selection stage in the study design was crucial in the classification performance of the learners.

#### 3.2.2. Classification Performed Using the DMI Dataset

First, classifications were performed on the 20% and 30% testing sets using all features. Then, 12 different scores were obtained for the variables using the XGBoost, LightGBM, CatBoost, AdaBoost, GBM, LR, and RF algorithms with the feature importance function; and the XGBoost, LightGBM, CatBoost, and GBM algorithms and the mutual info method in the SHAP functions. These scores were evaluated using the Borda method and the variables were ordered from the highest to the lowest based on their explanatory values. By using the top 10% (top 18 variables), 20% (top 36 variables), 30% (top 54 variables), and 40% (top 72 variables) of the ranked variables, classifications were performed on the 20% and 30% testing sets. The accuracy values of all these classifications are given in Table 3.

Table 3 lists the accuracy values of the 10 ML algorithms used to classify students’ well-being for the DMI dataset. In the classification performed using all the variables, the CatBoost algorithm was the best classifier, with an accuracy of almost 78%. In the classification using the top 18 variables with the highest explanatory power obtained from the feature-selection process, the CatBoost algorithm reached an approximately 75.5% accuracy on both the 30% and 20% testing sets. The accuracy value was only about 2.5% higher when using all the variables instead of the top 18 variables. However, the difference in other variable selection algorithms was very small. The CatBoost algorithm was the best in almost all of the classification models while the DT performed poorly in all classifications (see Table 3).

### 3.3. Important Variables

**Figure 2:**
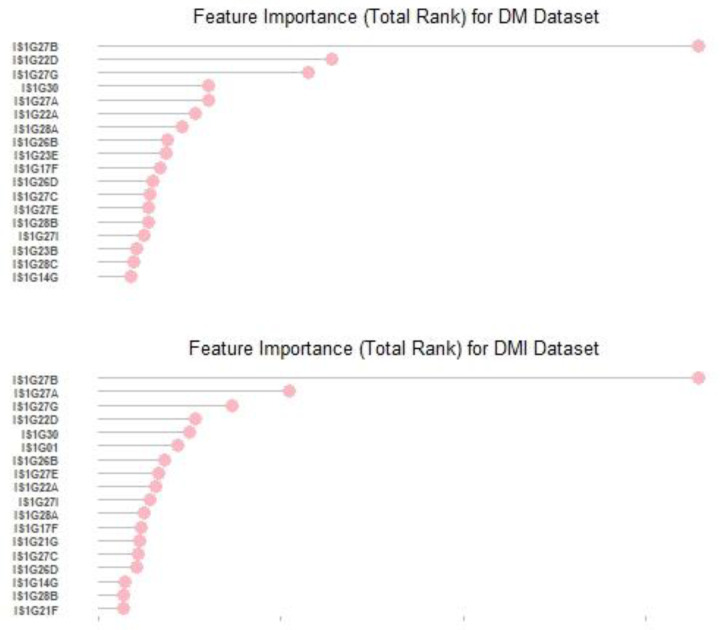
The top 10% influential factors for DM and DMI datasets.

After evaluating the classification results for learners, we investigated the relative importance of the variables. Overall, 178 variables were analyzed in this study. Figure 2 presents the top 10% influential factors based on the Borda total rank scores.

The feature-selection process in both datasets obtained the most influential variables on student well-being. Using the top 18 (10%) or the top 36 (20%) variables returned almost the same accuracy results as using all the features. The top 18 or the top 36 variables were very influential in classifying the well-being of students below or above the average. In other words, the remaining 60% of the variables were not as influential in the classification procedure.

For the DM and the DMI datasets, the highest-ranked variable that affected the student’s well-being was “I worried a lot about catching COVID-19 at school”. The following most influential factors on student well-being were “It became more difficult to know how well I was progressing” and “I was excited to catch up with friends” for the DM dataset. As for the DMI dataset, “I was more motivated to learn when school reopened than at any other time” and “I was excited to catch up with friends” were the second and third most influential factors on student well-being. The top 10% of the variables associated with student well-being in both datasets are presented in Table 4 and Table 5. The complete list of all variables ordered according to the Borda method can be found in the Supplementary Materials (Table S2).

Among 178 variables, gender was ranked 23rd in the DM dataset and 35th in the DMI dataset. The least influential variables on the DM dataset were whether the variety of schoolwork the students were given changed, how much the student liked videoconferencing (e.g., using Zoom, MS Teams) with the entire class for part (but not all) of the normal lesson/period to communicate with the teacher and classmates, and whether the student used a computer or tablet device that was used only by themselves at home during the COVID-19 disruption. The least influential variables on the DMI dataset were whether the parents or guardians were available and could help with the schoolwork at home, how much additional help the student received from other people to use school computer systems (e.g., email and learning-management systems) for the schoolwork, and how much the student liked individual videoconferencing (e.g., using Zoom and MS Teams) to communicate with a teacher during the COVID-19 disruption.

## 4. Discussion

Using data-mining approaches, we analyzed factors that affected student well-being during the COVID-19 pandemic. First, an imputed dataset was created along with the original dataset to accomplish this goal. Next, the students’ well-being IRT scores were estimated using the items in the REDS student database and the students were classified into two categories: (1) students whose well-being score was below the average well-being score of all students in the database and (2) students whose well-being score was above the average well-being score of all students in the database. Then, both single and ensemble learners were used to identify the most critical factors that affected the well-being of students during the COVID-19 pandemic.

We used two datasets in this study: the original and imputed data. The purpose here was to classify the outcome with the least number of variables, reveal the importance of these variables, and find the algorithm that performed the best classification. Similar accuracy values were obtained when the same testing sample sizes were compared in the classification processes performed using both datasets. Since most ML algorithms do not work when the data contain missing values, using imputed data allowed us to work with ML algorithms and make detailed comparisons. In this study, the CatBoost algorithm generally performed the best and the other boosting algorithms gave similar results using both datasets: classification performances varied between 76% and 78%.

We revealed the factors that affected eighth-grade students’ well-being in different countries. The present research provided insights into the factors that affected student well-being during the unprecedented times of the COVID-19 pandemic. An international database spanning several continents was used to investigate student well-being in eight countries. The most influential variables on student well-being during the COVID-19 outbreak were listed. We found that students generally had a fear of acquiring COVID-19 at school. We found limited research on adolescents’ concerns about COVID-19; however, this result was in line with the findings that worry regarding the COVID-19 pandemic was one of the variables that significantly predicted university students’ depression, anxiety, and stress based on the data that was collected at the end of the spring 2020 semester [65]. By looking at the database alone, we found that most students seemed to be worried about contracting COVID-19 when they returned to school: 87% of the students in the Burkina Faso sample, followed by 83% of the students in the Kenya sample and 74% of the students in the Ethiopia sample, strongly agreed or agreed that they worried a lot about contracting COVID-19 when returning to school after the pandemic’s disruption [36]. Since we explored several factors that affected student well-being during the COVID-19 outbreak, these factors, especially the most influential ones, should be taken into account to enhance the well-being state of students.

### Limitations

Although we used a rich and broad database, the data were collected from only eight countries, making it difficult to generalize our findings to other countries. In addition, this study was subject to recall and social desirability biases due to the nature of the survey data. Moreover, our findings should be interpreted cautiously since our analyses addressed prediction or association, not causality. Finally, we did not include other missing data imputation methods that may have led to different findings due to space limitations. Additionally, the REDS database has its own limitations, including a lack of an international monitoring program for quality control, the difference in the initial time of disruption in different countries, and low participation rates that were primarily due to the data-collection period that occurred during the COVID-19 outbreak [37].

## 5. Conclusions

Overall, this study found a significant level of worry among students about COVID-19, especially about contracting COVID-19 at school. Facing prolonged periods of living with uncertainty regarding their studies played a negative role in students’ well-being. Certain subgroups may require additional support, and the psychological impact of the virus can be far-reaching. Therefore, research teams should attempt to understand student well-being in these unprecedented times and beyond; policymakers should make informed decisions based on how youth are faring during the COVID-19 pandemic.

## Figures and Tables

**Table 1:** Well-being scores by country.

Country	*n (Total)*	Well-Being Score			
		DM		DMI	
		Mean	*SD*	Mean	*SD*
Burkina Faso	2450	0.67	0.85	−0.47	0.87
Denmark	1308	−0.47	0.73	0.03	0.87
Ethiopia	3613	0.26	0.99	0.39	0.92
Kenya	1594	−0.01	0.83	−0.23	0.89
Russian Federation	3502	−0.36	0.82	0.24	1.01
Slovenia	2494	−0.47	0.87	−0.11	0.85
United Arab Emirates	2849	0.01	0.86	−0.35	0.82
Uzbekistan	2910	0.15	0.76	0.15	0.77

**Table 2:** Classification accuracy for the DM dataset.

	Accuracy (%)									
Number of Variables (%)	178 (100%)		18 (10%)		36 (20%)		54 (30%)		72 (40%)	
Test Sample Size	30%	20%	30%	20%	30%	20%	30%	20%	30%	20%
CatBoost	77.06	76.81	75.92	76.23	76.27	77.39	77.32	76.91	77.69	77.58
LightGBM	76.72	77.05	75.19	76.01	76.14	77.08	76.64	76.71	76.99	77.09
XGBoost	75.85	75.85	74.79	75.09	75.42	76.09	76.45	76.83	76.59	76.30

**Table 3:** Classification accuracy for the DMI dataset.

	Accuracy (%)									
Number of Variables (%)	178 (100%)		18 (10%)		36 (20%)		54 (30%)		72 (40%)	
Test Sample Size	30%	20%	30%	20%	30%	20%	30%	20%	30%	20%
CatBoost	77.93	77.80	75.60	75.41	76.88	76.86	77.48	76.91	77.64	77.65
LightGBM	76.29	76.42	75.32	74.50	76.24	76.28	76.30	76.11	76.79	76.50
XGBoost	76.51	74.83	73.83	73.46	76.24	76.23	76.63	76.09	76.42	76.86
GBM	76.32	75.70	74.90	74.57	75.77	75.53	76.05	75.70	76.19	75.53
AdaBoost	74.97	74.35	74.36	74.25	74.61	74.74	74.89	74.71	74.53	74.57
*k*-NN	67.89	67.93	71.83	71.60	72.68	72.73	71.38	71.45	69.82	69.93
DT	66.80	65.30	65.75	66.07	65.93	66.77	66.94	68.10	66.44	67.35
RF	76.90	76.26	74.82	74.74	76.91	75.92	77.11	76.67	77.08	76.88
LR	73.70	73.46	73.30	73.12	73.33	73.19	73.81	73.75	73.65	73.26
SVM	76.54	76.47	75.02	75.07	76.45	76.16	76.54	76.26	76.42	76.52

**Table 4:** Top 10% most influential variables for the DM dataset.

Rank	Feature	Item
1	IS1G27B	I worried a lot about catching COVID-19 at school.
2	IS1G22D	It became more difficult to know how well I was progressing.
3	IS1G27G	I was excited to catch up with friends.
4	IS1G30	Overall, how prepared do you feel for learning from home if your school building closed for an extended period in the future?
5	IS1G27A	I was more motivated to learn when school reopened than at any other time.
6	IS1G22A	I learned about as much as before the COVID-19 disruption.
7	IS1G28A	I understood the changed arrangements in my school.
8	IS1G26B	Our family had to be more careful with money than usual.
9	IS1G23E	Health advice about COVID-19
10	IS1G17F	I was happy to be at home.
11	IS1G26D	One or both of my parents/guardians were stressed about their job.
12	IS1G27C	I found it hard to manage the COVID-19 routines at school (e.g., wearing a mask, social distancing)
13	IS1G27E	I felt that I had fallen behind in my learning compared to other students.
14	IS1G28B	My teachers went over the work we did during the COVID-19 disruption.
15	IS1G27I	My teachers seemed more caring towards me than they were before the COVID-19 disruption.
16	IS1G23B	Looking after my personal safety
17	IS1G28C	We rushed through a lot of new schoolwork.
18	IS1G14G	I found it difficult to get extra or different types of work from my teachers.

**Table 5:** Top 10% most influential variables for the DMI dataset.

Rank	Feature	Item
1	IS1G27B	I worried a lot about catching COVID-19 at school.
2	IS1G27A	I was more motivated to learn when school reopened than at any other time.
3	IS1G27G	I was excited to catch up with friends.
4	IS1G22D	It became more difficult to know how well I was progressing.
5	IS1G30	Overall, how prepared do you feel for learning from home if your school building closed for an extended period in the future?
6	IS1G01	Where did you attend school lessons during the COVID-19 disruption?
7	IS1G26B	Our family had to be more careful with money than usual.
8	IS1G27E	I felt that I had fallen behind in my learning compared to other students.
9	IS1G22A	I learned about as much as before the COVID-19 disruption.
10	IS1G27I	My teachers seemed more caring towards me than they were before the COVID-19 disruption.
11	IS1G28A	I understood the changed arrangements in my school.
12	IS1G17F	I was happy to be at home.
13	IS1G21G	My teachers encouraged me to learn.
14	IS1G27C	I found it hard to manage the COVID-19 routines at school (e.g., wearing a mask, social distancing)
15	IS1G26D	One or both of my parents/guardians were stressed about their job.
16	IS1G14G	I found it difficult to get extra or different types of work from my teachers.
17	IS1G28B	My teachers went over the work we did during the COVID-19 disruption.
18	IS1G21F	I had a good relationship with my teachers.

## Data Availability

The datasets analyzed for this study can be found in the Data & Tools section of the International Association for the Evaluation of Educational Achievement website: https://www.iea.nl/data-tools/repository/reds (accessed on 31 March 2022).

## Supplementary Materials

The following are available online at https://www.mdpi.com/article/10.3390/ijerph191811267/s1, Table S1: REDS student questionnaire; Table S2: The importance levels of the variables ordered according to the Borda method.
