# Supplementary material for: Exploring Factors That Affected Student Well-Being during the COVID-19 Pandemic: A Comparison of Data-Mining Approaches
Source: Int J Environ Res Public Health. 2022 Sep 7;19(18):11267. doi: 10.3390/ijerph191811267 (PMC9517244; doi:10.3390/ijerph191811267)
Supplement: Supplementary file 1 [file ijerph-19-11267-s001.zip › ijerph-1822562-SI.pdf]

Table S1. REDS student questionnaire.

Question	Item	Description
Q1	IS1G01	Where did you attend school lessons during the COVID-19 disruption?
Q2	-	During the COVID-19 disruption, did you have access to the following ICT devices at home?
	IS1G02A	Desktop or laptop computers
	IS1G02B	Tablet devices (e.g. iPad, Tablet PC)
	IS1G02C	Smart phones
Q3	IS1G03	During the COVID-19 disruption, did you have access to the internet at home?
Q4	-	In comparison with the time before the COVID-19 disruption, how much did your use of the following resources for schoolwork change?
	IS1G04A	Desktop or laptop computers
	IS1G04B	Tablet devices (e.g. iPad, Tablet PC)
	IS1G04C	Smart phones
	IS1G04D	Internet
	IS1G04E	Printed resources (e.g., text books, library books, work sheets)
	IS1G04F	Lessons provided as radio broadcasts
	IS1G04G	Lessons provided as television broadcasts
Q5	-	During the COVID-19 disruption, which of the following devices did you use at home?
	IS1G05A	A computer or tablet device used only by me
	IS1G05B	A computer or tablet device that was shared with others in my home
	IS1G05C	A smart<br>phone
Q6	IS1G06	During the COVID-19 disruption, how much of the schoolwork you completed at home was done using pen/pencil and paper?
Q7	-	Before, during and after the COVID-19 disruption, how well could you do each of the following tasks?
	IS1G07A	Connect a device to the internet
	IS1G07B	Send and receive email
	IS1G07C	Log into my school portal or learning system
	IS1G04D	Take photos, or record video or audio of my learning
	IS1G07E	Manage settings on a device to access school software
	IS1G07F	Send photos, videos or audio of my learning
	IS1G07G	Seesaw and Google classrooms to communicate with my teacher
	IS1G07H	Maintain devices (e.g. Update apps, check storage space)
	IS1G07I	Fix problems when my device is not working properly
	IS1G07J	Sending and receiving text, audio or video messages (e.g. using WhatsApp
Q8	IS1G08	Think about the computer applications (software) that you used in your lessons and schoolwork during the COVID-19 disruption
Q9	-	During the COVID-19 disruption, how often did you use the following forms of communication with your teachers and classmates?
	IS1G09A	Videoconferencing (e.g. using Zoom, MS Teams) with the whole class for an entire lesson/period
	IS1G09B	Videoconferencing (e.g. using Zoom, MS Teams) with the whole class for part (but not all) of my normal lesson/period
	IS1G09C	Videoconferencing (e.g. using Zoom, MS Teams) with my teacher and small groups of students
	IS1G09D	Sharing ideas about our schoolwork with other students using a computer
	IS1G09E	Individual videoconferencing (e.g. Using Zoom, MS Teams) with a teacher

	IS1G09F	Communicating with a teacher using emails
	IS1G09G	Having a phone conversation with a teacher
	IS1G09H	Receiving materials on the computer that I needed to print (e.g. worksheets or reference material)
	IS1G09I	Receiving printed materials from my school (e.g. worksheets or reference material)
	IS1G09J	Receiving short instructional videos (e.g. how to complete a practical task or an explanation of a topic)
	IS1G09K	Receiving information/materials related to my schoolwork through Radio or TV
	IS1G09L	Communicating with a teacher using a messaging service such as WhatsApp
Q10	-	During the COVID-19 disruption, how much did you like using each of the following forms of communication with your teachers and classmates?
	IS1G10A	Videoconferencing (e.g. using Zoom, MS Teams) with the whole class for an entire lesson/period
	IS1G10B	Videoconferencing (e.g. using Zoom, MS Teams) with the whole class for part (but not all) of my normal lesson/period
	IS1G10C	Videoconferencing (e.g. using Zoom, MS Teams) with my teacher and small groups of students
	IS1G10D	Sharing ideas about our schoolwork with other students using a computer
	IS1G10E	Individual videoconferencing (e.g. Using Zoom, MS Teams) with a teacher
	IS1G10F	Communicating with a teacher using emails
	IS1G10G	Having a phone conversation with a teacher
	IS1G09H	Receiving materials on the computer that I needed to print (e.g. worksheets or reference material)
	IS1G10I	Receiving printed materials from my school (e.g. worksheets or reference material)
	IS1G10J	Receiving short instructional videos (e.g. how to complete a practical task or an explanation of a topic)
	IS1G10K	Receiving information/materials related to my schoolwork through Radio or TV
	IS1G10L	Communicating with a teacher using a messaging service such as WhatsApp
Q11	-	During the COVID-19 disruption, how often did you receive materials for your lessons in the following ways?
	IS1G11A	They were delivered to me by post, school staff or another member of the community (e.g. Another parent)
	IS1G11B	I collected them directly from the school
	IS1G11C	I used a school-based online platform e.g. Compass, Seesaw
	IS1G11D	I received them by email
	IS1G11E	They were sent by other online methods (e.g. Through Google Drive, Microsoft One Drive)
	IS1G11F	I received them through a messaging service such as WhatsApp
Q12	-	During the COVID-19 disruption, how often did you do the following activities related to your schoolwork?
	IS1G12A	Research on the internet
	IS1G12B	Work from textbooks
	IS1G12C	Performance (dancing, role play) or physical activities
	IS1G12D	Activities where I used common household items to build models or experiments
	IS1G12E	Revision of work from lessons completed before the COVID-19 disruption
	IS1G12F	Working with other students
	IS1G12G	Asking for help from parents/guardians, other adults or other members of my family
Q13	-	During the COVID-19 disruption, how often did you use the following learning resources when doing schoolwork or attending lessons?
	IS1G13A	Tutorial software or practice programs
	IS1G13B	Computer-based information resources (e.g. websites, wikis, encyclopaedia)
	IS1G13C	Interactive digital learning resources (e.g. learning games or applications)

	IS1G13D	Digital textbooks
	IS1G13E	Printed textbooks
	IS1G13F	Printed worksheets
Q14	-	To what extent do you agree or disagree with the following statements about your learning during the COVID-19 disruption?
	IS1G14A	I found it hard to understand the schoolwork my teachers gave me
	IS1G14B	I learned more studying at home than when attending regular lessons at school
	IS1G14C	I was able to complete my schoolwork without having to ask anyone to help me
	IS1G14D	I was unable to get help for my schoolwork from a teacher or school support staff
	IS1G14E	I needed other people at home to help me complete my schoolwork
	IS1G14F	I needed to contact my classmates to help me complete my schoolwork
	IS1G14G	I found it difficult to get extra or different types of work from my teachers
	IS1G14H	I only did the work that I had to submit to my teacher
	IS1G14I	I got higher grades than before the COVID-19 disruption
Q15	-	During the COVID-19 disruption, who was available to help you with your schoolwork at home?
	IS1G15A	My parent(s) or guardian(s) was/were available and could help me with my schoolwork
	IS1G15B	Older siblings were available and could help me with my schoolwork
	IS1G15C	People other than my parents/guardians or siblings were available and could help me with my schoolwork
	IS1G15D	There was no one available who could help me with my schoolwork
Q16	-	During the COVID-19 disruption, how much additional help did you receive with your schoolwork from other people?
	IS1G16A	Help to find or access my schoolwork on computer
	IS1G16B	Help to plan my schoolwork for the day
	IS1G16C	Explanations of my schoolwork when needed
	IS1G16D	Study advice or tips
	IS1G16E	Help to do research
	IS1G16F	Encouragement to stay on task
	IS1G16G	Help to use the school computer systems (e.g. email, learning management system)
	IS1G16H	Teaching of additional skills that were not part of my school assignments
Q17	-	For how much of the time during the COVID-19 disruption do each of the following statements apply to your learning at home?
	IS1G17A	I had a quiet space to work with a desk and chair
	IS1G17B	I felt safer at home than I usually feel at school
	IS1G17C	I had to look after my siblings
	IS1G17D	I had to look after a sick or elderly relative
	IS1G17E	I felt I did not have the things I needed to complete my schoolwork at home
	IS1G17F	I was happy to be at home
	IS1G17G	I missed meals at home because I rely on the meals/breakfast/lunch offered at school
Q18	-	During the COVID-19 disruption, did the following aspects of your schoolwork change?
	IS1G18A	My motivation to complete my schoolwork
	IS1G18B	The variety of schoolwork I was given

	IS1G18C	My capacity to plan the completion of my schoolwork
	IS1G18D	The quality of my schoolwork
	IS1G18E	The amount of help I got from my parents/other family members with my schoolwork
	IS1G18F	The amount of distraction whilst completing my schoolwork
	IS1G18G	My ability to keep up with my schoolwork
	IS1G18H	My confidence in completing schoolwork
	IS1G18I	My contribution to class discussions
	IS1G18J	My skills in accessing online learning content
Q19	-	Think about your workload in a typical school week during the COVID-19 disruption. How much work did you submit to teachers in the learning areas below?
	IS1G19A	Language Arts: test language
	IS1G19B	Language arts: foreign or other national languages
	IS1G19C	Mathematics Add any appropriate national examples
	IS1G19D	Sciences (general science and/or physics, chemistry, biology, geology, earth sciences, technical science)
	IS1G19E	Human sciences / Humanities /Social Studies (history, geography, civics, law, economics, etc.)
	IS1G19F	Creative arts (visual arts, music, dance, drama, etc.)
	IS1G19G	Information technology, computer studies or similar
	IS1G19H	Practical and vocational subjects Add any appropriate national examples
	IS1G19I	Other (e.g. moral/ethics, physical education, personal and social development)
Q20	-	
	IS1G20A	Spoken feedback given to me individually
	IS1G20B	Spoken feedback given to small groups
	IS1G20C	Spoken feedback given to the whole class
	IS1G20D	Scores/grades
	IS1G20E	Written feedback on my work
	IS1G20F	Written or spoken feedback recorded through the school-based learning management apps e.g. Google Classroom, Seesaw
Q21	-	To what extent do you agree or disagree with the following statements about the support you received from your teachers during the COVID-19 disruption?
	IS1G21A	My teachers were available when I needed their help
	IS1G21B	My teachers made it clear how to best contact them
	IS1G21C	My teachers gave me feedback that I could understand
	IS1G21D	My teachers made a special effort to keep in contact with me
	IS1G21E	My teachers showed interest in my learning
	IS1G21F	I had a good relationship with my teachers
	IS1G21G	My teachers encouraged me to learn
	IS1G21H	My teachers adapted my schoolwork to meet my individual needs
Q22	-	
	IS1G22A	I learned about as much as before the COVID-19 disruption
	IS1G22B	I made more progress in some subjects than before the COVID-19 disruption
	IS1G22C	It became more difficult to use my teachers' feedback to improve my work
	IS1G22D	It became more difficult to know how well I was progressing
Q23	-	During the COVID-19 disruption, did your school or teachers give you information on the topics listed below to support your well-being?

	IS1G23A	Looking after my emotional wellbeing (e.g. relaxation methods)
	IS1G23B	Looking after my personal safety
	IS1G23C	Healthy eating
	IS1G23D	How to find people who can provide well-being advice
	IS1G23E	Health advice about COVID-19
	IS1G23F	Healthy working habits (such as taking breaks when studying)
	IS1G23G	Maintaining physical fitness
Q24	-	To what extent do you agree or disagree with the following statements about how you felt during the COVID-19 disruption?
	IS1G24A	I felt anxious about the changes in my schooling
	IS1G24B	I felt overwhelmed by what was happening in the world due to the COVID-19 pandemic
	IS1G24C	I felt overwhelmed by what was happening in my local area due to the COVID-19 pandemic
	IS1G24D	I was worried about how the disruption affected my learning
	IS1G24E	I was worried about how this disruption will affect my future education
	IS1G24F	I missed my usual contact with my classmates
	IS1G24G	I felt supported by my school
	IS1G24H	I had one or more teachers whom I felt comfortable to ask for help
	IS1G24I	I could not get my usual level of support from non-teaching support staff
	IS1G24J	I still felt part of the school
	IS1G24K	I was worried about catching COVID-19
	IS1G24L	My classmates were supportive of each other
	IS1G24M	I found it difficult to concentrate on my schoolwork
Q25	-	To what extent do you agree or disagree with the following statements about your well-being during the COVID-19 disruption?
	IS1G25A	I exercised (including walking) more than usual
	IS1G25B	I was able to do more of my usual outside of school activities scouts, guides, training for sports
	IS1G25C	I felt fit and healthy
	IS1G25D	I felt more lonely than usual
	IS1G25E	I got upset over things that would not have normally bothered me
	IS1G25F	I felt angry more often than usual
	IS1G25G	I did not feel like contacting my friends
	IS1G25H	I was more worried than usual about my friends and family getting sick
	IS1G25I	I used social media a lot more than before the COVID-19 disruption
	IS1G25J	I did not sleep as well as before the COVID-19 disruption
Q26	-	Were you affected by any of the following situations during the COVID-19 disruption?
	IS1G26A	One or both of my parents/ guardians lost their job
	IS1G26B	Our family had to be more careful with money than usual.
	IS1G26C	One or both of my parents/ guardians had to work from home
	IS1G26D	One or both of my parents/ guardians were stressed about their job
	IS1G26E	Our family had to move to live in a new location
	IS1G26F	I had to live away from my parents/ guardians

	IS1G26G	I had to help more at home with household tasks
Q26a	-	Do you do receive support from your school and teachers for a disability?
Q26b	-	Before the COVID-19 disruption, how much support was your school giving you in the following areas?
	IS1G26bA	How I move around: lifting and carrying things, holding things, moving from place to place, getting around your community
	IS1G26bB	How I learn: seeing, hearing, learning to read, learning to write, learning maths
	IS1G26bC	How I communicate: understanding what people say, telling people what I need, speaking clearly, having conversations
	IS1G26bD	How I take care of myself: personal grooming, using the bathroom, eating and drinking, and keeping myself safe
	IS1G26bE	How I relate to others – making friends, keeping friends, managing disagreements, understanding other points of view
	IS1G26bF	How I solve problems – solving problems, persisting, staying on task, completing tasks independently, coping with setbacks
Q26c	-	How did the support you received from your school in the following areas change during the COVID-19 disruption?
	IS1G26cA	How I move around: lifting and carrying things, holding things, moving from place to place, getting around your community
	IS1G26cB	How I learn: seeing, hearing, learning to read, learning to write, learning maths
	IS1G26cC	How I communicate: understanding what people say, telling people what I need, speaking clearly, having conversations
	IS1G26cD	How I take care of myself: personal grooming, using the bathroom, eating and drinking, and keeping myself safe
	IS1G26cE	How I relate to others – making friends, keeping friends, managing disagreements, understanding other points of view
	IS1G26cF	How I solve problems – solving problems, persisting, staying on task, completing tasks independently, coping with setbacks
Q27	-	To what extent do you agree or disagree with the following statements about your learning at school after the COVID-19 disruption?
	IS1G27A	I was more motivated to learn when school reopened than at any other time
	IS1G27B	I worried a lot about catching COVID-19 at school
	IS1G27C	I found it hard to manage the COVID-19 routines at school (e.g. wearing a mask, social distancing)
	IS1G27D	I found it hard to concentrate during class time
	IS1G27E	I felt that I had fallen behind in my learning compared to other students
	IS1G27F	I had to complete more assessments than usual
	IS1G27G	I was excited to catch up with friends
	IS1G27H	My classmates were friendlier than before the COVID-19 disruption
	IS1G27I	My teachers seemed more caring towards me than they were before the COVID-19 disruption
	IS1G27J	I felt safe at school
Q28	-	To what extent do you agree or disagree with the following statements about your school experiences after the COVID-19 disruption?
	IS1G28A	I understood the changed arrangements in my school
	IS1G28B	My teachers went over the work we did during the COVID-19 disruption
	IS1G28C	We rushed through a lot of new schoolwork
	IS1G28D	My class was less well-behaved than before the COVID-19 disruption
	IS1G28E	Staff from my school other than my teacher came to my class to ask about our well-being
	IS1G28F	We were reminded that school counsellors and support officers were available for individual appointments
	IS1G28G	Extra tuition was available to catch up on schoolwork
	IS1G28H	It was easier for me to access free lunches and/or breakfast than before the COVID-19 disruption
Q29	-	How confident are you now about the following aspects of your schoolwork?
	IS1G29A	Using videoconferencing software such as Zoom or MS Teams
	IS1G29B	Finding learning resources on my own
	IS1G29C	Planning when to do schoolwork on my own
	IS1G29D	Completing schoolwork independently
	IS1G29E	Assessing my learning progress

	IS1G29F	Seeking assistance from others when I need it
	IS1G29G	Using a learning management system or school learning platform (e.g. Seesaw)
Q30	IS1G30	Overall, how prepared do you feel for learning from home if your school building closed for an extended period in the future?
Q31	-	When were you born?
	IS1G31A	Month
	IS1G31B	Year
Q32	IS1G32	Are you a girl or a boy?
Q33	-	Do any of these people live at home with you most or all of the time?
	IS1G33A	Parent 1/Guardian 1 (for example, step-parent or foster parent)
	IS1G33B	Parent 2/Guardian 2 (for example, step-parent or foster parent)
	IS1G33C	Older siblings/or same age siblings (brothers or sisters including step-brothers and step-sisters)
	IS1G33D	Younger siblings (brothers or sisters including step-brothers and step-sisters)
	IS1G33E	Grandparents
	IS1G33F	Others
Q33a	IS1G33aA	What best describes you?
Q34	IS1G34	What language do you speak at home most of the time?
Q35	-	How many of the following ICT devices are currently used in your home?
	IS1G35A	Desktop or laptop computers
	IS1G35B	Tablet devices (e.g. iPad, Tablet PC)
	IS1G35C	Smart phones
Q36	-	Do you have any of these things at your home?
	IS1G36A	Study desk/table for your use
	IS1G36B	Books of your very own (do not count your school books)
	IS1G36C	Your own room
	IS1G36D	Internet connection
	IS1G36E	country-specific indicator of wealth
	IS1G36F	country-specific indicator of wealth
	IS1G36G	country-specific indicator of wealth
	IS1G36H	country-specific indicator of wealth
	IS1G36I	country-specific indicator of wealth
	IS1G36J	country-specific indicator of wealth
Q36	IS1G36	About how many books are there in your home?
Q37	IS1G37	What kind of work does your parent or guardian 1 do for his/her main job?
Q38	IS1G38	What is the highest level of education completed by your parent or guardian 1?
Q39	IS1G39	What kind of work does your parent or guardian 2 do for his/her main job?
Q40	IS1G40	What is the highest level of education completed by your parent or guardian 2?

Table S2. The importance levels of the variables ordered according to the Borda method.

Rank	Variable (DM)	Variable (DMI)	Rank	Variable (DM)	Variable (DMI)	Rank	Variable (DM)	Variable (DMI)
1	IS1G27B	IS1G27B	61	IS1G29B	IS1G28D	121	IS1G20A	IS1G09J
2	IS1G22D	IS1G27A	62	IS1G40	IS1G23C	122	IS1G12D	IS1G11B
3	IS1G27G	IS1G27G	63	IS1G03	IS1G17C	123	IS1G33A	IS1G13B
4	IS1G30	IS1G22D	64	IS1G18J	IS1G26C	124	IS1G15D	IS1G07E
5	IS1G27A	IS1G30	65	IS1G16F	IS1G19B	125	IS1G09H	IS1G13F
6	IS1G22A	IS1G01	66	IS1G06	IS1G14F	126	IS1G17E	IS1G05B
7	IS1G28A	IS1G26B	67	IS1G02C	IS1G20C	127	IS1G10H	IS1G21C
8	IS1G26B	IS1G27E	68	IS1G28G	IS1G04E	128	IS1G09F	IS1G09K
9	IS1G23E	IS1G22A	69	IS1G29D	IS1G10J	129	IS1G16C	IS1G16C
10	IS1G17F	IS1G27I	70	IS1G21D	IS1G21B	130	IS1G20D	IS1G20E
11	IS1G26D	IS1G28A	71	IS1G16E	IS1G17E	131	IS1G09B	IS1G12F
12	IS1G27C	IS1G17F	72	IS1G36	IS1G29B	132	IS1G14C	IS1G23A
13	IS1G27E	IS1G21G	73	IS1G17C	IS1G21A	133	IS1G21C	IS1G04B
14	IS1G28B	IS1G27C	74	IS1G21E	IS1G23D	134	IS1G09G	IS1G18D
15	IS1G27I	IS1G26D	75	IS1G21B	IS1G19C	135	IS1G02A	IS1G19E
16	IS1G23B	IS1G14G	76	IS1G18C	IS1G10I	136	IS1G09C	IS1G16H
17	IS1G28C	IS1G28B	77	IS1G17D	IS1G14D	137	IS1G19E	IS1G38
18	IS1G14G	IS1G21F	78	IS1G12C	IS1G29C	138	IS1G07F	IS1G09D
19	IS1G21G	IS1G22C	79	IS1G21A	IS1G07H	139	IS1G09J	IS1G13C
20	IS1G14B	IS1G07D	80	IS1G19I	IS1G28F	140	IS1G18D	IS1G19A
21	IS1G22C	IS1G23E	81	IS1G26A	IS1G20D	141	IS1G10G	IS1G09G
22	IS1G34	IS1G04C	82	IS1G04A	IS1G17D	142	IS1G09A	IS1G17A
23	IS1G32	IS1G14B	83	IS1G23F	IS1G04A	143	IS1G16H	IS1G07I
24	IS1G07D	IS1G23B	84	IS1G35A	IS1G35A	144	IS1G33E	IS1G02A
25	IS1G27H	IS1G29F	85	IS1G20C	IS1G14H	145	IS1G21H	IS1G09E
26	IS1G29F	IS1G28C	86	IS1G13F	IS1G18E	146	IS1G07C	IS1G13D
27	IS1G18F	IS1G04D	87	IS1G28F	IS1G18C	147	IS1G11D	IS1G20A
28	IS1G22B	IS1G27H	88	IS1G04B	IS1G36	148	IS1G15A	IS1G07F
29	IS1G29A	IS1G14I	89	IS1G13A	IS1G12D	149	IS1G13C	IS1G09F
30	IS1G16D	IS1G34	90	IS1G15C	IS1G14C	150	IS1G02B	IS1G10F
31	IS1G18A	IS1G18F	91	IS1G38	IS1G09H	151	IS1G19B	IS1G09B
32	IS1G21F	IS1G29A	92	IS1G33F	IS1G02C	152	IS1G07I	IS1G05C
33	IS1G23G	IS1G28H	93	IS1G01	IS1G16E	153	IS1G12A	IS1G10C
34	IS1G27D	IS1G18A	94	IS1G09E	IS1G08	154	IS1G18G	IS1G18I
35	IS1G28H	IS1G32	95	IS1G09I	IS1G29E	155	IS1G05C	IS1G10D
36	IS1G37	IS1G03	96	IS1G27J	IS1G15C	156	IS1G11A	IS1G33D
37	IS1G07G	IS1G14A	97	IS1G16B	IS1G12G	157	IS1G05B	IS1G05A
38	IS1G18H	IS1G40	98	IS1G20B	IS1G29D	158	IS1G18I	IS1G15D
39	IS1G04C	IS1G22B	99	IS1G14H	IS1G12A	159	IS1G10E	IS1G33B
40	IS1G23C	IS1G14E	100	IS1G10J	IS1G07G	160	IS1G09D	IS1G33E
41	IS1G14A	IS1G28E	101	IS1G11B	IS1G23F	161	IS1G16G	IS1G18G
42	IS1G07A	IS1G19H	102	IS1G08	IS1G19F	162	IS1G17A	IS1G19D
43	IS1G14E	IS1G27D	103	IS1G12F	IS1G12C	163	IS1G13E	IS1G09A
44	IS1G04E	IS1G07A	104	IS1G10C	IS1G20B	164	IS1G17B	IS1G10H
45	IS1G26E	IS1G28G	105	IS1G19F	IS1G13A	165	IS1G35B	IS1G16B
46	IS1G27F	IS1G26E	106	IS1G07H	IS1G10A	166	IS1G10A	IS1G10K
47	IS1G10I	IS1G07C	107	IS1G12G	IS1G26F	167	IS1G13D	IS1G13E
48	IS1G14F	IS1G12E	108	IS1G13B	IS1G26A	168	IS1G33D	IS1G11A
49	IS1G04D	IS1G23G	109	IS1G20E	IS1G35C	169	IS1G15B	IS1G16A
50	IS1G28D	IS1G16D	110	IS1G14D	IS1G09I	170	IS1G07E	IS1G15B

51	IS1G35C	IS1G06	111	IS1G10D	IS1G21H	171	IS1G19D	IS1G11D
52	IS1G23D	IS1G18H	112	IS1G26C	IS1G07B	172	IS1G16A	IS1G02B
53	IS1G19H	IS1G16F	113	IS1G19C	IS1G12B	173	IS1G10K	IS1G33F
54	IS1G29C	IS1G37	114	IS1G23A	IS1G10B	174	IS1G33B	IS1G33A
55	IS1G12E	IS1G27F	115	IS1G09K	IS1G21D	175	IS1G07B	IS1G35B
56	IS1G14I	IS1G27J	116	IS1G26F	IS1G17B	176	IS1G05A	IS1G10E
57	IS1G28E	IS1G33C	117	IS1G19A	IS1G09C	177	IS1G10B	IS1G16G
58	IS1G39	IS1G18J	118	IS1G18E	IS1G18B	178	IS1G18B	IS1G15A
59	IS1G33C	IS1G39	119	IS1G10F	IS1G10G			
60	IS1G29E	IS1G21E	120	IS1G12B	IS1G19I			
